# 4,6-Dimethyl-2-*p*-tolyl­pyrimidine

**DOI:** 10.1107/S160053680904210X

**Published:** 2009-10-17

**Authors:** Chen Xu, Zhi-Qiang Wang, Fei-Fei Cen, Lin Cheng, Bao-Ming Ji

**Affiliations:** a College of Chemistry and Chemical Engineering, Luoyang Normal University, Luoyang 471022, People’s Republic of China; b Chemical Engineering and Pharmaceutics School, Henan University of Science and Technology, Luoyang 471003, People’s Republic of China

## Abstract

The mol­ecule of the title compound, C_13_H_14_N_2_, is located on a crystallographic mirror plane. The aromatic rings make a dihedral angle of 3.4 (2)°. The H atoms of the methyl groups on the benzene ring are disordered over two positions; their site-occupation factors were fixed at 0.5. In the crystal, inter­molecular C—H⋯π contacts form infinite chains perpendicular to the *b* axis.

## Related literature

The title compound was derived from the reaction of *p*-tolylmercutic chlorides and 4,6-dimethyl-2-iodopyrimidine. For general background to the use of organomercury compounds in cross-coupling reactions, see: Beletskaya *et al.* (2001 ▶); Braga *et al.* (2004 ▶). For a related structure, see: Santoni *et al.* (2008 ▶). For the synthesis, see: Xu *et al.* (2009*a* ▶,*b* ▶).
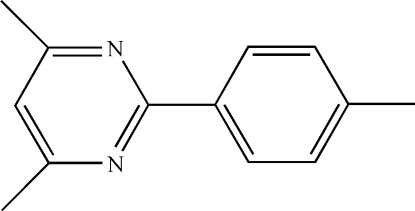


## Experimental

### 

#### Crystal data

C_13_H_14_N_2_
*M* *_r_* = 198.26
Orthorhombic, 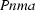

*a* = 7.2086 (10) Å
*b* = 12.4668 (18) Å
*c* = 12.4335 (18) Å
*V* = 1117.4 (3) Å^3^
*Z* = 4
Mo *K*α radiation
μ = 0.07 mm^−1^
*T* = 296 K
0.35 × 0.25 × 0.22 mm

#### Data collection

Bruker SMART APEX CCD area-detector diffractometer
Absorption correction: multi-scan (*SADABS*; Sheldrick, 1996 ▶) *T*_min_ = 0.976, *T*_max_ = 0.985
7934 measured reflections
1089 independent reflections
777 reflections with *I* > 2σ(*I*)
*R*_int_ = 0.025

#### Refinement

*R*[*F* ^2^ > 2σ(*F* ^2^)] = 0.042
*wR*(*F* ^2^) = 0.131
*S* = 1.06
1089 reflections
78 parameters
H-atom parameters constrained
Δρ_max_ = 0.19 e Å^−3^
Δρ_min_ = −0.14 e Å^−3^

### 

Data collection: *SMART* (Bruker, 2004 ▶); cell refinement: *SAINT* (Bruker, 2004 ▶); data reduction: *SAINT*; program(s) used to solve structure: *SHELXS97* (Sheldrick, 2008 ▶); program(s) used to refine structure: *SHELXL97* (Sheldrick, 2008 ▶); molecular graphics: *SHELXTL* (Sheldrick, 2008 ▶); software used to prepare material for publication: *SHELXL97* and *PLATON* (Spek, 2009 ▶).

## Supplementary Material

Crystal structure: contains datablocks global, I. DOI: 10.1107/S160053680904210X/si2211sup1.cif

Structure factors: contains datablocks I. DOI: 10.1107/S160053680904210X/si2211Isup2.hkl

Additional supplementary materials:  crystallographic information; 3D view; checkCIF report

## Figures and Tables

**Table 1:** Hydrogen-bond geometry (Å, °)

*D*—H⋯*A*	*D*—H	H⋯*A*	*D*⋯*A*	*D*—H⋯*A*
C8—H8⋯*Cg*1^i^	0.93	2.79	3.638 (2)	152

Symmetry code: (i) 
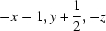
. *Cg*1 is the centroid of the pyrimidine ring.

**Fig. 1.**
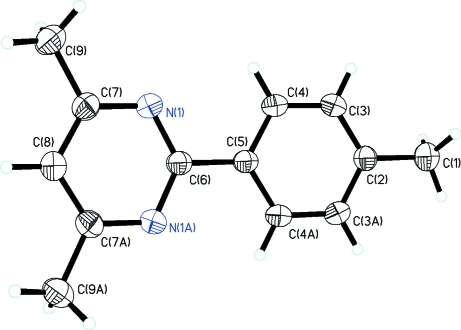
The molecular structure of the title compound with displacement ellipsoids at the 30% probability level, the disordered H atoms are omitted (Symmetry code A: -x + 2, -y, -z).

**Fig. 2.**
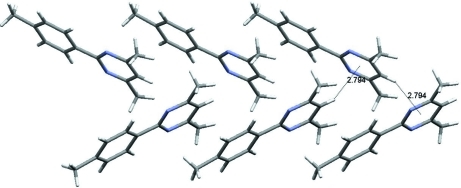
Partial view of the crystal packing showing the formation of the infinite chain of molecules formed by the C—H···π interactions.

## supplementary crystallographic information

### Comment

The organomercury compounds have a number of notable advantages over other organometallic compounds commonly used in cross-coupling reactions, including higher selectivity of reactions, extra stability and easy availability by a direct mercuration (Beletskaya *et al.*, 2001; Braga *et al.*, 2004). We have recently reported ferrocene-heterocycles were obtained from the coupling reaction(Xu *et al.*, 2009*a*,b). Here we report the crystal structure of the title compound, derived from the reaction of *p*-tolylmercutic chlorides and 4,6-dimethyl-2-iodopyrimidine.

Due to the molecular mirror symmetry m of the title compound (Fig.1), and coincidence with the crystallographic mirror plane m (space group *Pnma*),the atoms C1, C2, C5, C8, H8 are half occupied and the H atoms of the methyl groups in the benzene ring are disordered over two positions; their site-occupation factors were fixed at 0.5. The aromatic rings have very small angles between their planes (dihedral angle is 3.4 (2)°) due to the absence of H—H repulsion (Santoni *et al.*, 2008). Fig.2 shows that in the crystal there exist intermolecular C—H···π interactions (Table 1, *Cg*1 is the centroid of the pyrimidine ring).

### Experimental

The title compound was obtained from the coupling reaction of *p*-tolylmercutic chlorides and 4,6-dimethyl-2-iodopyrimidine as described in literature (Xu *et al.*, 2009*b*) and recrystallized from ethanol at room temperature to give the desired crystals suitable for single-crystal X-ray diffraction.

### Refinement

H atoms attached to C atoms of the title compound were placed in geometrically idealized positions and treated as riding with C—H distances constrained to 0.93–0.96 Å, and with *U*_iso_(H)=1.2–1.5*U*_eq_(C).

### Crystal data

**Table:**

C_13_H_14_N_2_	*D*_x_ = 1.179 Mg m^−^^3^
*M**_r_* = 198.26	Mo *K*α radiation, λ = 0.71073 Å
Orthorhombic, *P**n**m**a*	Cell parameters from 1634 reflections
*a* = 7.2086 (10) Å	θ = 2.3–23.3°
*b* = 12.4668 (18) Å	µ = 0.07 mm^−^^1^
*c* = 12.4335 (18) Å	*T* = 296 K
*V* = 1117.4 (3) Å^3^	Block, colourless
*Z* = 4	0.35 × 0.25 × 0.22 mm
*F*(000) = 424	

### Data collection

**Table:**

Bruker SMART APEX CCD area-detector diffractometer	1089 independent reflections
Radiation source: fine-focus sealed tube	777 reflections with *I* > 2σ(*I*)
graphite	*R*_int_ = 0.025
phi and ω scans	θ_max_ = 25.5°, θ_min_ = 2.3°
Absorption correction: multi-scan (*SADABS*; Sheldrick, 1996)	*h* = −8→8
*T*_min_ = 0.976, *T*_max_ = 0.985	*k* = −15→15
7934 measured reflections	*l* = −15→14

### Refinement

**Table:**

Refinement on *F*^2^	Primary atom site location: structure-invariant direct methods
Least-squares matrix: full	Secondary atom site location: difference Fourier map
*R*[*F*^2^ > 2σ(*F*^2^)] = 0.042	Hydrogen site location: inferred from neighbouring sites
*wR*(*F*^2^) = 0.131	H-atom parameters constrained
*S* = 1.06	*w* = 1/[σ^2^(*F*_o_^2^) + (0.0557*P*)^2^ + 0.291*P*] where *P* = (*F*_o_^2^ + 2*F*_c_^2^)/3
1089 reflections	(Δ/σ)_max_ < 0.001
78 parameters	Δρ_max_ = 0.19 e Å^−^^3^
0 restraints	Δρ_min_ = −0.14 e Å^−^^3^

### Special details

**Table:**

Geometry. All e.s.d.'s (except the e.s.d. in the dihedral angle between two l.s. planes)are estimated using the full covariance matrix. The cell e.s.d.'s are takeninto account individually in the estimation of e.s.d.'s in distances, anglesand torsion angles; correlations between e.s.d.'s in cell parameters are onlyused when they are defined by crystal symmetry. An approximate (isotropic)treatment of cell e.s.d.'s is used for estimating e.s.d.'s involving l.s. planes.
Refinement. Refinement of *F*^2^ against ALL reflections. The weighted *R*-factor *wR* andgoodness of fit *S* are based on *F*^2^, conventional *R*-factors *R* are basedon *F*, with *F* set to zero for negative *F*^2^. The threshold expression of*F*^2^ > σ(*F*^2^) is used only for calculating *R*-factors(gt) *etc*. and isnot relevant to the choice of reflections for refinement. *R*-factors basedon *F*^2^ are statistically about twice as large as those based on *F*, and *R*-factors based on ALL data will be even larger.

### Fractional atomic coordinates and isotropic or equivalent isotropic displacement parameters (Å^2^)

**Table:**

	*x*	*y*	*z*	*U*_iso_*/*U*_eq_	Occ. (<1)
C1	0.8988 (4)	0.2500	0.6728 (2)	0.0709 (8)	
H1A	0.8781	0.2870	0.7394	0.106*	0.50
H1B	0.9952	0.2856	0.6332	0.106*	0.50
H1C	0.9355	0.1774	0.6874	0.106*	0.50
C2	0.7231 (3)	0.2500	0.60770 (17)	0.0499 (6)	
C3	0.6383 (2)	0.34507 (13)	0.57708 (13)	0.0544 (5)	
H3	0.6934	0.4100	0.5953	0.065*	
C4	0.4741 (2)	0.34546 (12)	0.52012 (13)	0.0522 (5)	
H4	0.4201	0.4105	0.5009	0.063*	
C5	0.3884 (3)	0.2500	0.49107 (16)	0.0449 (5)	
C6	0.2078 (3)	0.2500	0.43367 (17)	0.0468 (5)	
C7	−0.0325 (2)	0.34504 (13)	0.36089 (13)	0.0529 (5)	
C8	−0.1188 (3)	0.2500	0.33466 (18)	0.0549 (6)	
H8	−0.2331	0.2500	0.3000	0.066*	
C9	−0.1170 (3)	0.45227 (14)	0.33570 (16)	0.0732 (6)	
H9A	−0.0974	0.5001	0.3951	0.110*	
H9B	−0.2477	0.4438	0.3235	0.110*	
H9C	−0.0598	0.4814	0.2724	0.110*	
N1	0.13292 (18)	0.34589 (10)	0.41062 (10)	0.0509 (4)	

### Atomic displacement parameters (Å^2^)

**Table:**

	*U*^11^	*U*^22^	*U*^33^	*U*^12^	*U*^13^	*U*^23^
C1	0.0703 (17)	0.0703 (18)	0.0720 (17)	0.000	−0.0169 (14)	0.000
C2	0.0541 (14)	0.0531 (13)	0.0425 (11)	0.000	−0.0007 (10)	0.000
C3	0.0600 (11)	0.0446 (9)	0.0584 (10)	−0.0043 (8)	−0.0036 (8)	−0.0048 (7)
C4	0.0588 (10)	0.0390 (9)	0.0589 (10)	0.0022 (7)	−0.0028 (8)	0.0002 (7)
C5	0.0507 (12)	0.0407 (11)	0.0433 (11)	0.000	0.0027 (10)	0.000
C6	0.0545 (13)	0.0430 (12)	0.0428 (11)	0.000	0.0018 (10)	0.000
C7	0.0550 (10)	0.0541 (10)	0.0496 (9)	0.0045 (8)	0.0009 (7)	0.0034 (7)
C8	0.0510 (13)	0.0608 (15)	0.0528 (13)	0.000	−0.0047 (11)	0.000
C9	0.0702 (12)	0.0598 (12)	0.0896 (14)	0.0091 (10)	−0.0128 (10)	0.0094 (10)
N1	0.0546 (8)	0.0449 (8)	0.0532 (8)	0.0031 (6)	−0.0025 (6)	0.0021 (6)

### Geometric parameters (Å, °)

**Table:**

C1—C2	1.503 (3)	C5—C6	1.485 (3)
C1—H1A	0.9600	C6—N1^i^	1.3426 (16)
C1—H1B	0.9600	C6—N1	1.3426 (16)
C1—H1C	0.9600	C7—N1	1.343 (2)
C2—C3^i^	1.387 (2)	C7—C8	1.378 (2)
C2—C3	1.387 (2)	C7—C9	1.502 (2)
C3—C4	1.380 (2)	C8—C7^i^	1.378 (2)
C3—H3	0.9300	C8—H8	0.9300
C4—C5	1.3885 (19)	C9—H9A	0.9600
C4—H4	0.9300	C9—H9B	0.9600
C5—C4^i^	1.3885 (19)	C9—H9C	0.9600
			
C2—C1—H1A	109.5	C4^i^—C5—C6	121.00 (11)
C2—C1—H1B	109.5	N1^i^—C6—N1	125.8 (2)
H1A—C1—H1B	109.5	N1^i^—C6—C5	117.07 (10)
C2—C1—H1C	109.5	N1—C6—C5	117.07 (10)
H1A—C1—H1C	109.5	N1—C7—C8	121.12 (16)
H1B—C1—H1C	109.5	N1—C7—C9	116.67 (15)
C3^i^—C2—C3	117.4 (2)	C8—C7—C9	122.20 (16)
C3^i^—C2—C1	121.28 (11)	C7—C8—C7^i^	118.7 (2)
C3—C2—C1	121.28 (11)	C7—C8—H8	120.7
C4—C3—C2	121.48 (16)	C7^i^—C8—H8	120.7
C4—C3—H3	119.3	C7—C9—H9A	109.5
C2—C3—H3	119.3	C7—C9—H9B	109.5
C3—C4—C5	120.81 (16)	H9A—C9—H9B	109.5
C3—C4—H4	119.6	C7—C9—H9C	109.5
C5—C4—H4	119.6	H9A—C9—H9C	109.5
C4—C5—C4^i^	118.0 (2)	H9B—C9—H9C	109.5
C4—C5—C6	121.00 (11)	C6—N1—C7	116.62 (15)
			
C3^i^—C2—C3—C4	1.2 (3)	C4^i^—C5—C6—N1	178.65 (17)
C1—C2—C3—C4	−178.02 (19)	N1—C7—C8—C7^i^	0.3 (3)
C2—C3—C4—C5	−0.3 (3)	C9—C7—C8—C7^i^	−179.77 (14)
C3—C4—C5—C4^i^	−0.7 (3)	N1^i^—C6—N1—C7	0.6 (3)
C3—C4—C5—C6	177.47 (16)	C5—C6—N1—C7	−178.53 (15)
C4—C5—C6—N1^i^	−178.65 (17)	C8—C7—N1—C6	−0.4 (3)
C4^i^—C5—C6—N1^i^	−0.6 (3)	C9—C7—N1—C6	179.62 (16)
C4—C5—C6—N1	0.6 (3)		

Symmetry codes: (i) *x*, −*y*+1/2, *z*.

### Hydrogen-bond geometry (Å, °)

**Table:**

*D*—H···*A*	*D*—H	H···*A*	*D*···*A*	*D*—H···*A*
C8—H8···Cg1^ii^	0.93	2.79	3.638 (2)	152

Symmetry codes: (ii) −*x*−1, *y*+1/2, −*z*.
